# Porous Si Microparticles Infiltrated with Magnetic Nanospheres

**DOI:** 10.3390/nano10030463

**Published:** 2020-03-04

**Authors:** Elena Chistè, Gloria Ischia, Marco Gerosa, Pasquina Marzola, Marina Scarpa, Nicola Daldosso

**Affiliations:** 1 Department of Computer Science, Fluorescence Laboratory, University of Verona, 37134 Verona, Italy; elena.chiste@univr.it; 2 Department of Industrial Engineering, University of Trento, 38123 Trento, Italy; gloria.ischia@unitn.it; 3 Department of Morphological-Biomedical Sciences, Section of Anatomy and Histology, University of Verona, 37134 Verona, Italy; marco.gerosa@univr.it (M.G.); pasquina.marzola@univr.it (P.M.); 4 Department of Physics, Laboratory of Nanoscience, University of Trento, 38123 Trento, Italy; marina.scarpa@unitn.it

**Keywords:** porous silicon microparticles, Super Paramagnetic Iron Oxide Nanoparticles (SPIONs), electron microscopy, MRI, theranostics

## Abstract

Porous silicon (pSi) microparticles obtained by porosification of crystalline silicon wafers have unique optical properties that, together with biodegradability, biocompatibility and absence of immunogenicity, are fundamental characteristics to candidate them as tracers in optical imaging techniques and as drug carriers. In this work, we focus on the possibility to track down the pSi microparticles also by MRI (magnetic resonance imaging), thus realizing a comprehensive tool for theranostic applications, i.e., the combination of therapy and diagnostics. We have developed and tested an easy, quick and low-cost protocol to infiltrate the COOH-functionalized pSi microparticles pores (tens of nanometers about) with magnetic nanospheres (SPIONs—Super Paramagnetic Iron Oxide Nanoparticles, about 5–7 nm) and allow an electrostatic interaction. The structural properties and the elemental composition were investigated by electron microscopy techniques coupled to elemental analysis to demonstrate the effective attachment of the SPIONs along the pores’ surface of the pSi microparticles. The magnetic properties were investigated under an external magnetic field to determine the relaxivity properties of the material and resulting in an alteration of the relaxivity of water due to the SPIONs presence, clearly demonstrating the effectiveness of the easy functionalization protocol proposed.

## 1. Introduction

Porous silicon (pSi) is a sponge-like material photoluminescent at room temperature. [1] Its distinctive properties make this material a promising tool for theranostics, i.e., the union of therapy and diagnostics. [2] In fact, it is biocompatible: [3] it degrades into silicic acids that kidneys are able to secrete, and it is neither toxic for the cells nor activator of the immune system. It could be exploited as bioimaging tracer in-vitro and in-vivo, due to its photoluminescence, [4,5] as carrier for drug loading and release, due to its porosity with large surface to volume ratio, [6,7] or as PTT (photothermal therapy) [8] or PDT (photodynamic therapy) [9] agent. Porous silicon is produced from crystalline silicon by a cheap and simple fabrication process, i.e., electrochemical etching in acid solution, resulting in pSi microparticles with photoluminescence (PL) in the orange-red portion of the visible spectrum [10,11] due to the quantum confinement effect. [12] The electrochemical etching produces pores on the silicon wafer surface, whose dimensions are adjustable with the etching parameters: in our optimized procedure, the pores are around 20–30 nm. [13] A hydrosilylation procedure (leading to a surface terminated with carboxyl-groups, which are negatively charged at pH > 4) is sufficient to stabilize the pSi microparticles for years in ethanol. [13] To homogenize the microparticles dimension and to reduce and manage their average size, we recently established a simple post-synthesis procedure, based on ultrasounds, without affecting the optical properties. [14]

One of the main concerns about the acceptance of this material in theranostics was the fast PL quenching in biological media. To overcome this issue, the pSi microparticles surface has been coated either with organic polymer (e.g., PEG or chitosan) by covalent attachment [15] or with inorganic atomic layer deposition (ALD) of TiO_2_ resulting in a shell with tunable thickness. [16] This allows the stabilization of the material and of its optical properties for several months. The functionalized microparticles were shown to be effective carriers of model drugs. [15]

The aim of the present work is to investigate the possibility of adding magnetic properties to the pSi microparticles to be traced by MRI (magnetic resonance imaging). MRI is a versatile and informative diagnostic technique, since the contrast in MRI is multifactorial, depending not only on proton density but also on T_1_ and T_2_ relaxation rates and flow. [17] Nanoparticles (NPs) with magnetic properties are widely used to further increase this contrast and therefore improve the biomedical diagnosis. [18,19] For magnetic NPs exploitation in theranostics, the magnetic remanence should vanish once the external magnetic field is switched off: that is one of the main reasons because, among all the magnetic NPs employed in MRI, SPIONs (SuperParamagnetic Iron Oxide Nanoparticles) are the most promising. [20,21] In fact, they are able to substantially modify the relaxation time of the interacting water molecules and are therefore used as contrast agents. [22,23] In particular, they belong to the class of negative contrast agents, meaning that they affect preferentially the transversal relaxation time T_2_ and consequently darken the MR image thus increasing the contrast between tissues. Furthermore, SPIONs biocompatibility [17] and the toxicity testing in animals [24] supports their use in biomedical applications.

Within this paper, we explored the possibility to fill the pores and decorate the pSi microparticles porous surface by magnetite (Fe_3_O_4_) nanoparticles with a dimension of few nm, with the goal of easily infiltrating the porous structure and favoring electrostatic attachment at the pSi surface. 

Few studies were previously reported about SPIONs infiltrated within the pores of porous silicon [25] or mesoporous silica nanoparticles. [26] In the former, the pSi microparticles were placed in an aqueous solution of magnetic NPs, which were infused inside the porous structure and embedded by a thermal oxidation/dehydration procedure, which is anyway long and requires special ovens. The magnetic pSi microparticles are drug loaded and delivered under the guidance of a magnetic field. In the latter, the magnetite NPs were anchored on the mesoporous silica nanoparticles by a boronate esters linker, to produce a pH-responsive tool for targeted delivery to low pH tissues.

The structural and magnetic results of this experimental work clearly show that, by means of an easy (just chemical mixing in a simple bucket), fast (only few minutes) and cheap (very inexpensive reagents: less than 80 euro-cents per mg) chemical protocol, based on the use of cyclohexane diamine (a small molecule that bears two positively charged groups) without the need of any oxidation steps, it is possible to get luminescent pSi microparticles infiltrated with SPIONs, which could be loaded in large quantity and electrostatically attached to the surface of the functionalized pSi pores. The absence of covalent binding enables a possible separation of the magnetic nanoparticles from the porous matrix for further MRI applications. Moreover, contrary to other procedures, it avoids the embedding of the SPIONs within a coating layer on the Si surface, thus opening the possibility of further molecular targeting. The use of the porous silicon matrix as a carrier for the SPIONs prevent the need of polymer’s shielding, [27] in perspective of in-vivo tests, and protect them from being cleaved in the circulation. In addition to that, in our approach, the photoluminescence of the functionalized pSi infiltrate with SPIONs is completely maintained. 

## 2. Materials and Methods 

Porous silicon samples have been fabricated according to the procedure already assessed in our laboratory, [13] which is briefly summarized in the following.

### 2.1. pSi Fabrication and Carboxyl Functionalization 

The porosification of boron-doped p-type Si wafers (<100> oriented, 10–20 Ω·cm resistivity, University wafers, Boston, MA, USA) was obtained by electrochemical etching at constant current (80 mA·cm^−2^) in ethanol: HF (16%) solution in a PTFE cell for 15 minutes. The pSi layer was removed from the wafer, dispersed in toluene and was fragmented into microparticles by 20 min sonication in a thermal bath. At the end of this procedure, microparticles with pore size about 20–30 nm, bearing Si-H groups on the surface are produced. [28] Light-driven hydrosilylation by acrylic acid in toluene at 50 °C under mild stirring (two hours) has been performed to introduce COOH groups at the porous surface. The obtained solution was washed (10 min centrifugation and removal of the supernatant) several times in ethanol and stored in this solvent. After this step, the microparticles bear COOH groups on the external surface and inside the pores, as previously proved by titled angle XPS. [29] However, some oxidation occurs and some SiO_x_ groups are present as shown by FTIR spectra. [28]

SPIONs (Super Paramagnetic Iron Oxide—Fe_3_O_4_—Nanoparticles) were purchased from Sigma-Aldrich, St. Louis, MO, USA (concentration: 5 mg·mL^−1^ in water, magnetization > 25 emu·g^−1^ at 4500 Oe, average particle size: 5–7 nm) and diluted in PBS to perform the experiments. We chose phosphate-buffered saline (PBS, pH 7.4) as a solvent in perspective of in-vitro tests. For the SPIONs functionalization (see in the following), the (±)-trans-1,2-Diaminocyclohexane (8.3 M) was purchased from Sigma-Aldrich (St. Louis, MO, USA).

### 2.2. Samples Characterization 

The structural and magnetic properties of the samples were studied by DLS (Dynamic Light Scattering), S/TEM (Scanning/Transmission Electron Microscopy) coupled to EDS (Energy Dispersive X-ray Spectroscopy) and MRI (Magnetic Resonance Imaging) measurements.

The surface charge (ζ-potential) of SPIONs and their size distribution, before and after diamine addition, was determined by Zetasizer Nano-SZ (Malvern) instrument, by dynamic light scattering (DLS) with a 633 nm laser beam. The measurements were performed at 25 °C after the dispersion of the SPIONs in PBS, pH 7.4, and 5 min of sonication in a thermal bath. 

TEM (Transmission electron microscopy) analysis was performed with an S/TEM ThermoFisher Talos F200S operating at 200 kV. The microscope is equipped with an integrated EDS (Energy Dispersive X-rays Spectroscopy) system with two windowless silicon drift detectors (SDD). The samples were observed in both TEM and STEM mode in order to investigate the SPIONs average size and the possible interaction between the magnetic nanoparticles and the pSi microparticles. Moreover, the STEM mode allows evaluating the actual elemental distribution in the sample collecting EDS maps. To support the samples during the observation, a 50 mL drop of this solution (in PBS and then water) was deposited on TEM copper support grids covered by a holey amorphous carbon film.

The magnetic properties were investigated by using a Pharmascan system operating at 7 T (Bruker, Germany). The longitudinal, T_1_, and transversal, T_2_, relaxation times were measured by acquiring an MSME (multi-spin multi-echo) pulse sequence. It consists of a 90° excitation RF pulse, followed by repeated 180° refocusing pulses, separated by a constant interval or echo-time. The signals coming from each point of the images were mediated to obtain the relaxation curve decays, that were fitted with an exponential decay function to obtain the T_1_ and T_2_ values. Afterwards, the longitudinal and transversal relaxivities r_1_ and r_2_ were determined by varying the sample concentration. The values obtained for the pSi-SPIONs sample were compared to the relaxivity of SPIONs and that of pSi-COOH microparticles alone.

### 2.3. SPIONs Infiltration within the pSi-COOH Microparticles 

The key step of the sample preparation was the decoration of pSi-COOH microparticles with the purchased iron oxide nanoparticles (SPIONs). We infiltrated them into the pores and covered the pSi surface with the NPs by electrostatic interaction. Since both the materials are negatively charged, we functionalized the SPIONs surface with a molecule bearing positively charged groups to favor the attachment of the nanoparticles onto the porous surface of the microparticles. The scheme of the sample preparation is reported in Figure S1, in the ESI.

We chose a diamine ((±)-trans-1,2-Diaminocyclohexane) to functionalize the iron oxide nanoparticles to let them become positively charged. The diamine was protonated by the addition of HCl to let the terminal amino group (NH_2_) become positive (NH_3_^+^). Then, the cyclohexane diamine was added to the magnetic nanospheres (0.2 mg·mL^−1^) in PBS and the NPs dimension and surface charge were adjusted by varying the diamine concentration from 0.3 M to 2 M. Thus, the diamine-functionalized SPIONs were incubated with 0.6 mg·mL^−1^ pSi-COOH microparticles. SPIONs decorated pSi microparticles (labelled as pSi-SPIONs microparticles) were re-dispersed in water: the sample was centrifuged, the surnatant was removed and then refilled with fresh water several times. This step was also important to assess if the nanoparticles were or not attached to the pSi microparticles. 

## 3. Results and Discussion

### 3.1. SPIONs Characterization and Functionalization 

**Figure 1:**
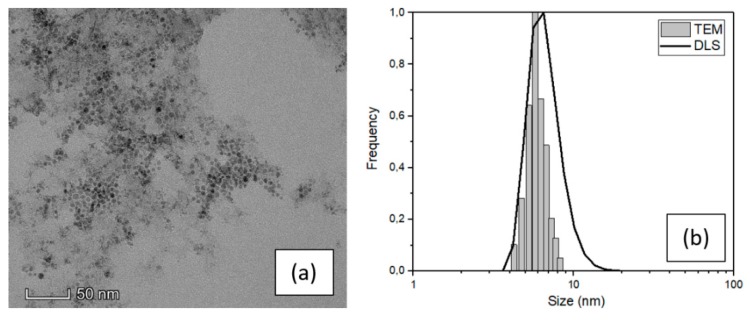
(**a**) TEM image and (**b**) size distribution of SPIONs dispersed in water obtained by TEM and DLS.

The structural properties of magnetite nanoparticles (SPIONs) were analyzed by TEM and DLS techniques to obtain the real average size and the size distribution. Figure 1a shows a characteristic TEM image of the SPIONs in water and Figure 1b the comparison between the size distributions obtained by TEM and DLS techniques.

The SPIONs particles showed in the TEM image (Figure 1) feature spherical shape and narrow distribution of dimension highly less than 10 nm in diameter. A high-resolution image of a single particle is reported in the ESI (Figure S2a) with the diffraction pattern relative to the agglomerate of Figure 1a, which demonstrates that the nanoparticles are composed of magnetite. [30] An evaluation of their dimension was carried out using ImageJ software and processing a fair number of TEM images. The result of this analysis leads to an average value of (6 ± 2) nm (see the histogram in Figure 1b) which is consistent with the nominal value of the purchased NPs and confirm the narrow size distribution. The average SPIONs size was also investigated by DLS, with an average value of (7 ± 3) nm which is consistent with the TEM result, although slightly overestimated because the DLS measurement is performed in liquid and gives an estimation of the hydrodynamic diameter. [31]

**Figure 2:**
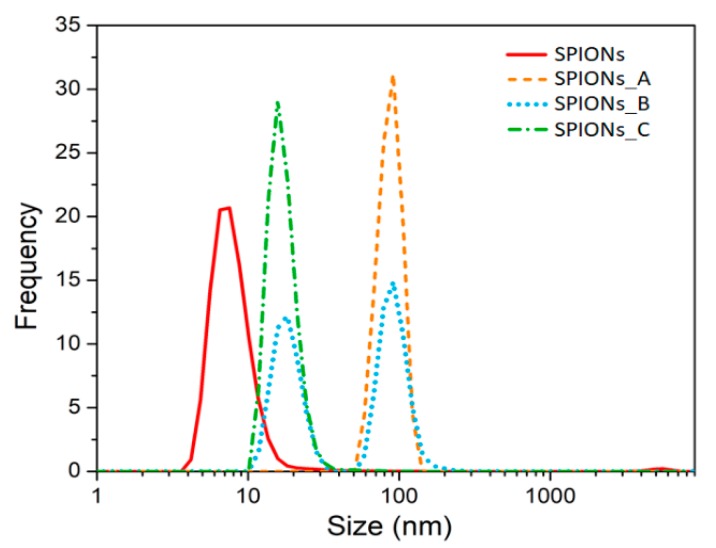
Size distribution obtained by DLS technique of diluted SPIONs and after the functionalization with increasing cyclohexane diamine concentration. Each distribution is the average of three measurements.

Then, the surface charge (i.e., ζ-potential value) was determined by DLS measurement and the surface charge distribution of the purchased SPIONs was found to be very broad and peaked at about −25–30 mV (see Figure S3a in ESI), in contrast with the declared neutral charge of the purchased magnetic nanospheres. Since the pSi microparticles are negatively charged too, they would repulse the SPIONs. Hence, we studied and validated a simple chemical procedure to make them positive: a functionalization with cyclohexane diamine molecules as described in detail in the experimental section. We investigated the effect of different cyclohexane diamine concentration on the ζ-potential value (see Figure S3 in ESI) and on the average size distribution (Figure 2) of the functionalized SPIONs (namely sample SPIONs_A, SPIONs_B and SPIONs_C) as the cyclohexane diamine is 0.3 M, 1 M and 2 M, respectively.

After the addition of the protonated cyclohexane diamine to SPIONs, we realized that the size distribution (orange dashed line of Figure 2) was surprisingly centered at about 100 nm. This is due to the agglomeration of the SPIONs because of an almost equal amount of both positive and negative charged SPIONs. In fact, a broad and peaked charge distribution has been found (see Figure S3 in the ESI) suggesting that only a partial functionalization was achieved for such a concentration of cyclohexane diamine.

To avoid the agglomeration and to increase the amount of positively charged SPIONs, we increased the ratio between protonated cyclohexane diamines and SPIONs, first by a factor three (sample SPIONs_B) and we obtained a size distribution with two populations (see blue dotted line in Figure 2): one peak is still centered at about 100 nm and it is related to the agglomerated particles, the other peak is at about 20 nm and it is related to the positively charged SPIONs. Thus, we further increase the ratio of cyclohexane diamine to SPIONs up to 10 mmol/mg (see green line in Figure 2) thus decreasing the average size distribution up to (18 ± 3) nm.

These results are in line with the ζ−potential results that clearly show that SPIONs_C has a positive surface charge (see panel (c) of Figure S3 in the ESI).

With these experimental findings, we set up a protocol to get positively charged SPIONs to the pSi-COOH microparticles, whose morphology and porosity have been carefully characterized in previously published works. [14,28] The resulting sample (namely pSi-SPIONs microparticles) was dispersed in PBS.

### 3.2. pSi-SPIONs Structural Properties 

**Figure 3:**
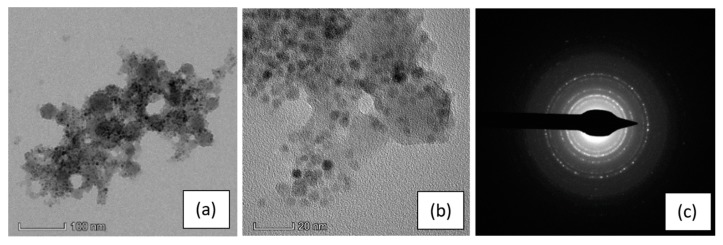
TEM images of a pSi microparticle after SPIONs infiltration at: (**a**) 100 nm scale and (**b**) 20 nm scale; (**c**) the SAED (selected area electron diffraction) pattern indexed as magnetite (PDF card 82–1533).

To verify that functionalized SPIONs were effectively infiltrated onto the pSi microparticles, we performed TEM analysis: the results are reported in Figure 3. 

Figure 3a shows a pSi microparticle, with its porous structure and a size of about 400 nm, whose surface is covered by SPIONs (dark dots in the picture), clearly on the porous structure. Their presence is even more evident in the magnified image (panel b) and evidenced by the diffraction pattern generated from the SPIONs (panel c). The ring pattern is consistent with the SPIONs dimensions and indicates a random orientation of the Fe_3_O_4_ nanocrystal. It is worth noting that the diffraction pattern of Si crystallites, produced from electrochemical etching, is hardly detectable, due to their random orientation and their very small dimension, with a large amount of voids. [32]

The presence of the SPIONs onto the pSi microparticles was also confirmed by EDS elemental analysis (see Figure S4 in the ESI) performed on the microparticle showed in panel (a) of Figure 3, indeed, the EDS spectrum exhibit the peaks relative to the characteristic elements of SPIONs and pSi microparticles.

**Figure 4:**
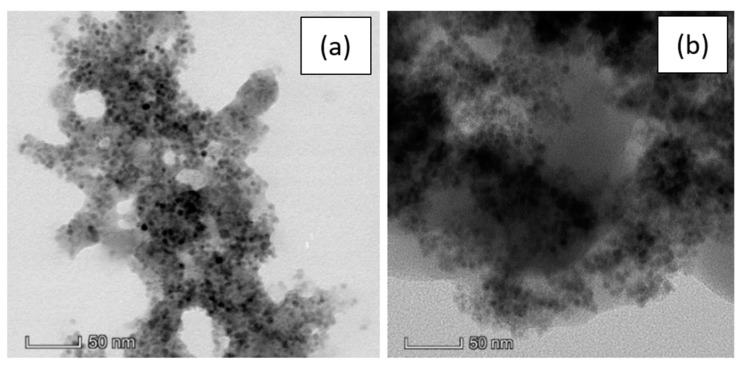
TEM images of a pSi microparticle after SPIONs decoration at 50 nm scale in: (**a**) PBS; (**b**) water.

The TEM observations were repeated after the re-dispersion in water and a comparison between the two solvents, PBS and water, is reported in Figure 4.

Here we can observe no substantial difference between the pSi-SPIONs microparticles in PBS (Figure 4a) or water (Figure 4b). The morphology is not modified by the re-dispersion, but the PBS buffer gives more contrast to the image and, consequently, the SPIONs are more visible. 

**Figure 5:**
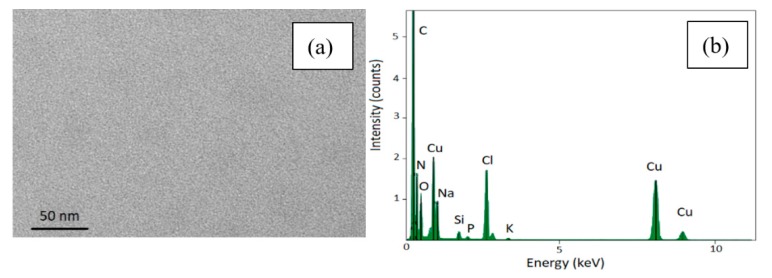
TEM image of the supernatant removed after centrifugation (panel **a**) and its EDS spectrum (panel **b**).

The surnatant obtained from the washing to re-disperse the sample in water was collected and probed by TEM and no SPIONs have been observed at all similarly to DLS investigation of the surnatant. This suggests that almost the totality of the functionalized SPIONs is infiltrated and electrostatically attached to the porous surface meaning even after several centrifugations and washing cycles (see Figure 5a). Besides the structural and morphological analysis, we also performed the EDS characterization of the supernatant without observing any peaks related to neither Fe_3_O_4_ nor porous silicon (see Figure 5b).

**Figure 6:**
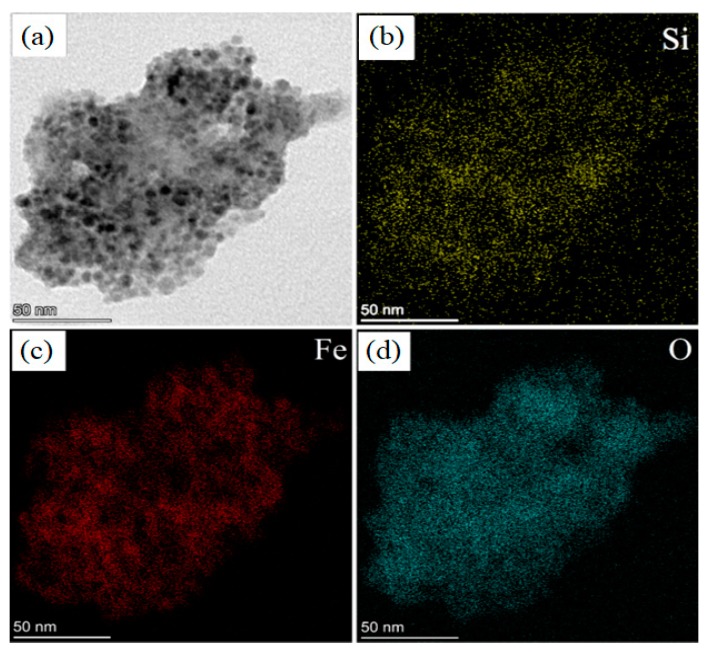
STEM (BF) images of: (**a**) a pSi microparticle after SPIONs decoration at 50 nm scale; elemental mapping of: (**b**) Si; (**c**) Fe; (**d**) O.

**Figure 7:**
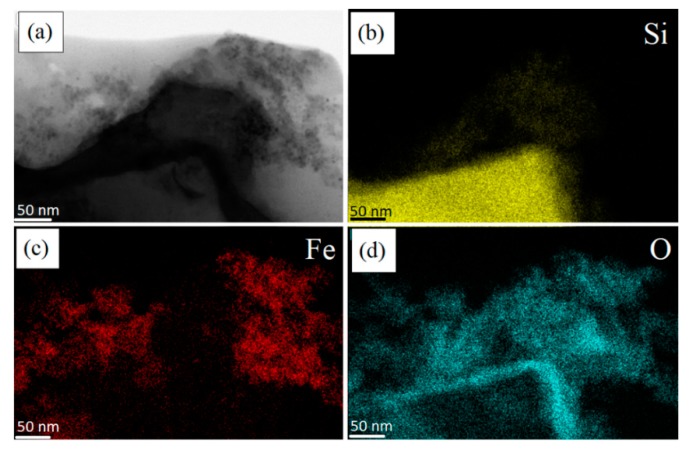
STEM images of: (**a**) a non-porous pSi microparticle after SPIONs decoration at 50 nm scale; elemental mapping of: (**b**) Si; (**c**) Fe; (**d**) O.

The sample was observed also in STEM mode, in which a focalized electron beam is scanned on the sample surface allowing to combine morphological investigation with EDS mapping. It is a powerful technique to investigate the actual elemental distribution inside the material with high spatial resolution. The results of STEM analysis are reported in Figure 6 and Figure 7.

Figure 6 shows the comparison between STEM Bright Field (BF) image (panel a) and elemental maps of Si (panel b), Fe (panel c) and O (panel d). This is very convenient to understand if the SPIONs are on the porous surface: we observed a quite perfect overlap between the distribution of Si (panel b), Fe (panel c) and O atoms (panel d). 

On the other hand, we performed STEM maps on a fragment of non-porous silicon, see the portion of the triangle at the bottom of Figure 7a: it shows a flat surface and homogeneous intensity. The small piece of crystalline silicon is characterized by a more compact structure (see the Si “yellow dots” in panel b with respect to those of Figure 6b) and its border is covered by a SiO_2_ layer, i.e., the cyan line clearly visible in panel (d). 

As it can be observed, in this case, the SPIONs do not attach onto silicon, due to the absence of porosity and proper surface functionalization due to previous surface oxidation. In fact, the positions of Fe atoms (panel c) and Si atoms (panel b) are not superimposable, meaning that the binding between nonporous silicon and SPIONs was not possible. In this case, a cloud of SPIONs (Fe and O atoms) are accumulated at the right top of the images, not at the non-porous Si surface.

### 3.3. pSi-SPIONs Magnetic Properties 

**Figure 8:**
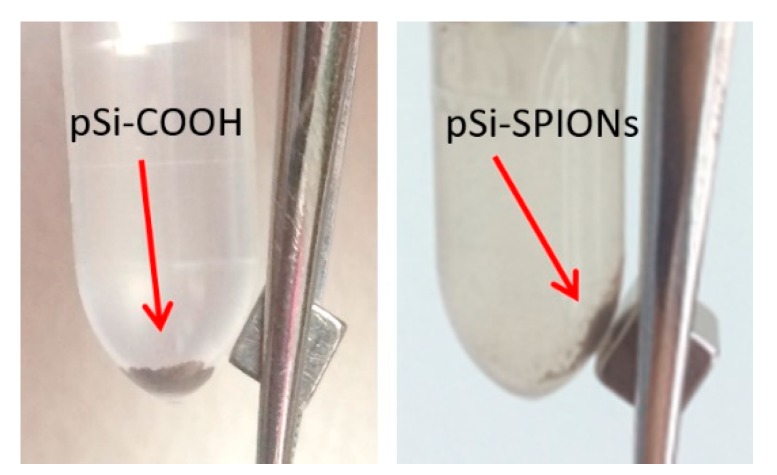
Evidence of the pSi-SPIONs microparticles, highlighted by an arrow, magnetic properties, differently from pSi-COOH microparticles, not attracted by the magnet.

After having proved the effectiveness of the functionalization protocol and of the infiltration procedure, we looked at the magnetic properties of the “new” system. As first evidence, Figure 8 shows the proof that magnetic properties were added to the pSi microparticles by SPIONs decoration: the SPIONs decorated pSi microparticles are attracted by a magnet, differently from pSi-COOH microparticles.

It is worth reminding here that the diagnostic application of pSi microparticles infiltrate with SPIONs in MRI relies on their capability to affect the water relaxation times. The relaxivities of pSi microparticles decorated with SPIONs were therefore investigated by MRI technique. Both longitudinal (spin-lattice) relaxivity r_1_ and transversal (spin-spin) relaxivity r_2_ were determined. [33] The relaxivity is obtained by measuring the relaxation time of the sample at different NPs concentration and then extrapolated via linear fit by using the formula: 1/T_i_ = 1/T _i,0_ + r_i_ ∙ c.

**Figure 9:**
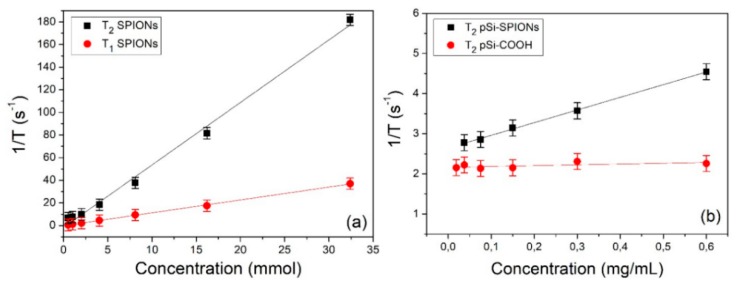
Relaxivity determination: (**a**) 1/T_1_ and 1/T_2_ relaxation rates of SPIONs as a function of the iron concentration; (**b**) 1/T_2_ relaxation rate of pSi-SPIONs and pSi-COOH microparticles as a function of the microparticles concentration.

First the longitudinal and transversal relaxivity of SPIONs in water were measured. The relaxation rates (1/T_i_) as a function of the iron concentration are shown in Figure 9a. Values of about 5.5 and 1.1 (mmol)^−1^s^−1^ are obtained for r_2_ and r_1_, respectively. These values are compatible with the literature and, as expected, the value of longitudinal relaxivity is substantially lower than that of transversal relaxivity [34]: r_2_/r_1_≈5. In fact, SPIONs are usually employed as negative contrast agents and act primarily on T_2_ relaxation time. 

In a second stage, we measured the relaxation efficacy of pSi-SPIONs and pSi microparticles in water (panel b); in this case, the relaxation rates have been reported as a function of the pSi concentration expressed in mg·mL^−1^ and therefore the obtained relaxivities are expressed in (mL·mg^−1^) s^−1^. Concerning the pSi microparticles (Figure 9b), we did not observe, within the experimental error, any variation of T_1_ and T_2_ as a function of the microparticles concentration. Nevertheless, a slight difference was found with respect to the relaxation times of pure water (T_1_ ≈ 3100 ms, T_2_ ≈ 600 ms), due to the interaction of water with the pSi-COOH microparticles. When pSi-SPIONs microparticles (Figure 9b) were added to water, transversal relaxivity was altered, while the effect on longitudinal relaxivity was negligible since T_1_ was found almost not to be concentration-dependent. The observed alteration in transversal relaxation deserves further investigations and paves the way for diagnostic applications of pSi-SPIONs in MRI. 

## 4. Conclusions

In this paper, we demonstrated the possibility to combine magnetic properties to the well-known properties of functionalized pSi microparticles, by infiltrating and decorating the pores with iron oxide nanoparticles (SPIONs). Thanks to an easy and fast chemical protocol based on the functionalization with cyclohexane diamine molecules, positively charged SPIONs are obtained, in a very short time and with a little cost. Furthermore, this new protocol enables a possible separation of the magnetic nanoparticles for further MRI applications and, contrary to other procedures, it avoids the embedding of the SPIONs within a coating layer on the Si surface. They can easily enter the pores (about 20 nm in size) because of their small dimension (about 6 nm) and attach to the functionalized porous silicon surface by electrostatic interactions. By TEM and STEM images analysis, we proved the magnetic nanospheres to be attached to the pSi surface. On the other hand, no attachment was observed in the case of non-porous functionalized surfaces (i.e., the small crystalline silicon portion). As first evidence of magnetic properties, we verified that the pSi microparticles decorated with SPIONs are effectively attracted by a magnet and that variations in the transversal relaxivity were observed, in comparison with “naked” pSi microparticles. Further studies will be carried out to optimize the relaxivity of the material by using other SPIONs and to quantitatively compare the relaxivity of pSi-SPIONs with that of other nanomaterials.

These encouraging new results pave the way to the exploitation of functionalized pSi microparticles infiltrated with magnetic nanospheres in the wide field of theranostics.

## Supplementary Materials

The following are available online at https://www.mdpi.com/2079-4991/10/3/463/s1: Figure S1: Functionalized porous Si microparticles infiltration scheme; Figure S2: HR-TEM image and diffraction pattern of a SPION nanoparticle; Figure S3: ζ-potential distribution of functionalized SPIONs; Figure S4: EDS spectrum of pSi-SPIONs microparticles.
