# Supplementary material for: Porous Si Microparticles Infiltrated with Magnetic Nanospheres
Source: Nanomaterials (Basel). 2020 Mar 4;10(3):463. doi: 10.3390/nano10030463 (PMC7153621; doi:10.3390/nano10030463)
Supplement: Supplementary file 1 [file nanomaterials-10-00463-s001.pdf]

Supplementary

Porous Si microparticles infiltrated with magnetic nanospheres

Elena Chistè [1], Gloria Ischia [2], Marco Gerosa [3], Pasquina Marzola [3], Marina Scarpa [4] and Nicola Daldosso [1,*]

[1] Department of Computer Science, Fluorescence Laboratory, University of Verona, 37134, Italy; elena.chiste@univr.it
[2] Department of Industrial Engineering, University of Trento, 38123, Italy; gloria.ischia@unitn.it;
[3] Department of Morphological-Biomedical Sciences, Section of Anatomy and Histology, University of Verona, 37134, Italy; marco.gerosa@univr.it (M.G.); pasquina.marzola@univr.it (P.M.)
[4] Department of Physics, Laboratory of Nanoscience, University of Trento, 38123, Italy; marina.scarpa@unitn.it
* Correspondence: nicola.daldosso@univr.it

Received: 29 February 2020; Accepted: 03 March 2020; Published: date

LIST OF CONTENTS

The electronic supporting information (ESI) contains 4 figures:

Figure S1. Functionalized porous Si microparticles infiltration scheme: the yellow dots represent the SPIONs, the red cross the positively charged diamine and the pSi-COOH microparticles are in blue.

Figure S2. High resolution TEM image of a SPION nanoparticle, where the lattice planes are visible, (panel a) and diffraction pattern of the selected area (Figure 4) with the indexing relative to magnetite (panel b).

Figure S3. ζ-potential distribution obtained by DLS technique of diluted SPIONs (panel a) and after the functionalization with different diamine concentration (panel b and c).

Figure S4. EDS spectrum of pSi-SPIONs microparticles. The presence of C and Cu is linked to the TEM grid used to support the sample during the observation, the other element are characteristic of the sample.

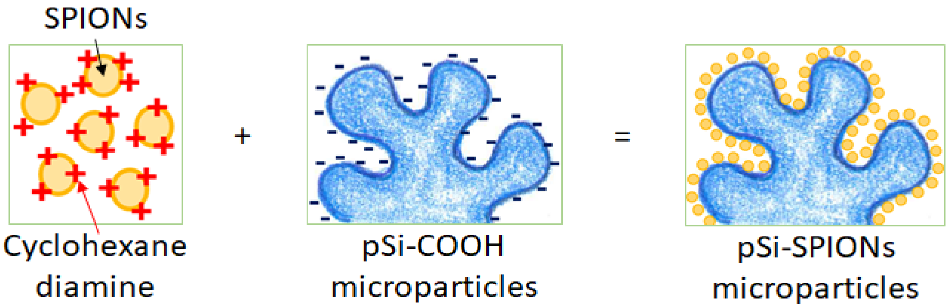

Figure S1. Functionalized porous Si microparticles infiltration scheme: the yellow dots represent the SPIONs, the red cross the positively charged diamine and the pSi-COOH microparticles are in blue.

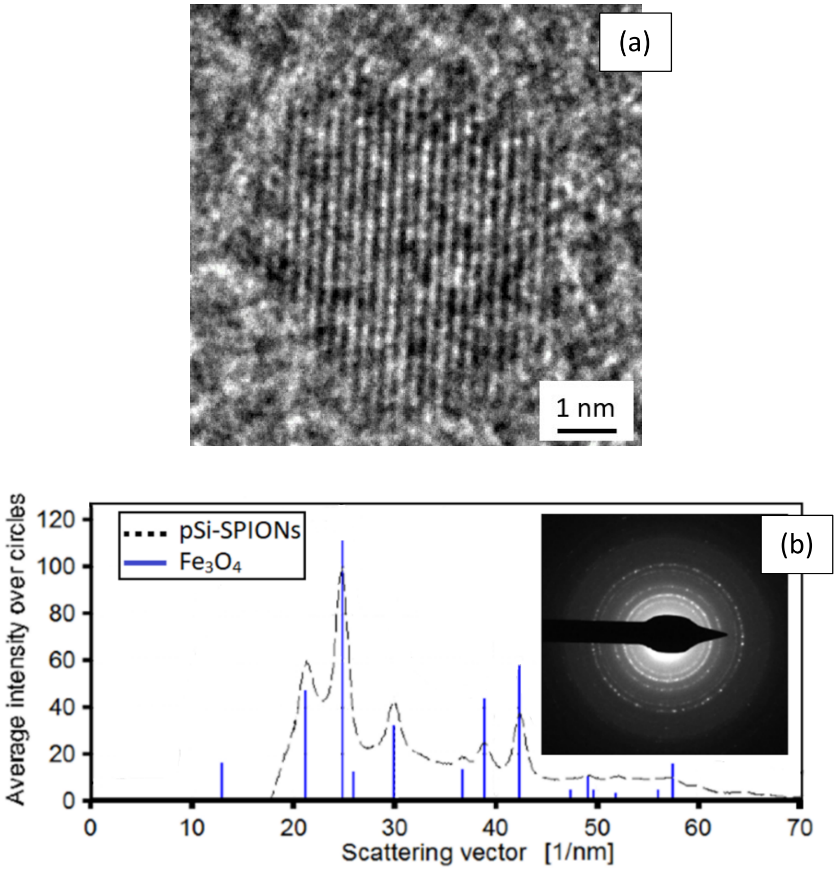

Figure S2. High resolution TEM image of a SPION nanoparticle, where the lattice planes are visible, (panel a) and diffraction pattern of the selected area (Figure 4) with the indexing relative to magnetite (panel b).

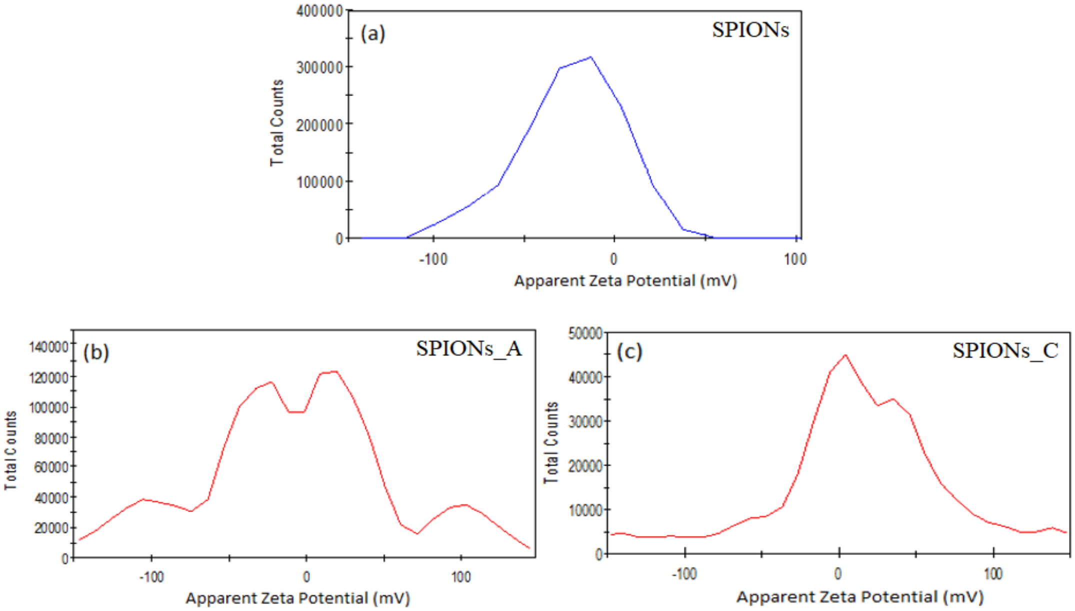

Figure S3. ζ-potential distribution obtained by DLS technique of diluted SPIONs (panel a) and after the functionalization with different diamine concentration (panel b and c).

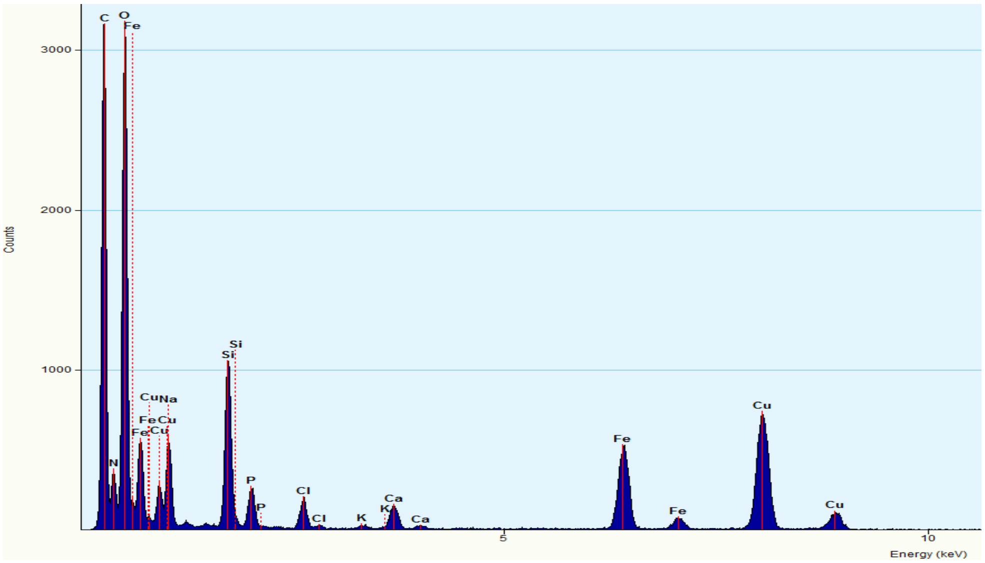

Figure S4. EDS spectrum of pSi-SPIONs microparticles. The presence of C and Cu is linked to the TEM grid used to support the sample during the observation, the other element are characteristic of the sample.
